# Glycemic assessment when hemoglobin A1c is unreliable: a clinical-laboratory framework for kidney disease, anemia, and hemoglobinopathies

**DOI:** 10.3389/fendo.2026.1898713

**Published:** 2026-09-01

**Authors:** Tianqiang Wu, Zihan Guan, Wenpin Cai

**Affiliations:** 1 Department of Endocrinology, Wenzhou TCM Hospital of Zhejiang Chinese Medical University, Wenzhou, Zhejiang, China; 2 Department of Laboratory Medicine, Wenzhou TCM Hospital of Zhejiang Chinese Medical University, Wenzhou, Zhejiang, China

**Keywords:** anemia, chronic kidney disease, continuous glucose monitoring, dialysis, glycemic discordance, glycemic monitoring, hemoglobin A1c, hemoglobinopathies

## Abstract

Hemoglobin A1c is central to diabetes diagnosis and monitoring, but its interpretation becomes clinically fragile when red-cell biology, hemoglobin composition, assay behavior, kidney failure, protein turnover, or glucose-data quality affects the assumptions behind the result. Existing reviews have described many causes of hemoglobin A1c-glycemia mismatch; the remaining practical problem is how clinicians and laboratories can choose a safer, decision-appropriate metric when the result is discordant with glucose data, symptoms, phenotype, or treatment response. This review synthesizes evidence from diabetes care, laboratory medicine, nephrology, hematology, and diabetes technology to propose a decision-oriented clinical-laboratory framework for selected high-risk settings: advanced chronic kidney disease and dialysis, anemia or altered red-cell turnover, and hemoglobin variants or hemoglobinopathies. The framework does not replace hemoglobin A1c with a single alternative marker. Instead, it asks which clinical decision is being made, whether hemoglobin A1c is biologically and analytically interpretable, whether comparator glucose data are adequate, and which adjunct metric is least likely to mislead in that scenario. Continuous glucose monitoring, structured self-monitoring, laboratory plasma glucose, glycated albumin, fructosamine, and selected secondary constructs are considered according to their decision value and failure modes. The proposed approach is an interpretive aid for clinician-laboratory communication, not a validated guideline, diagnostic algorithm, or therapeutic pathway.

## 1 Introduction: when a familiar marker requires contextual interpretation

Hemoglobin A1c (HbA1c) remains central to diabetes care because it summarizes longer-term glycemic exposure in a standardized laboratory result. Its usefulness, however, depends on assumptions that may be hidden at the point of care: hemoglobin needs to be an appropriate substrate, red-cell survival needs to reasonably match the intended glycation window, the assay needs to fit the hemoglobin context, and the result needs to match the clinical decision. When these assumptions fail, HbA1c may not be reliable enough to guide diagnosis, monitoring, treatment intensification, treatment deintensification, or hypoglycemia surveillance on its own (1, 2).

The same boundary applies to population-derived translations between HbA1c and mean glucose and to long-term outcome evidence; the clinical aim is accurate assessment of glycemic exposure and risk, not uncritical reliance on a surrogate when that surrogate is biologically or analytically compromised (3, 4).

The unresolved problem is translational rather than descriptive. Broad reviews have already summarized HbA1c-glycemia mismatch across biological, analytical, and clinical settings, and recent data on HbA1c-glucose management indicator (GMI) discordance have provided checklist approaches for interpreting disagreement between laboratory HbA1c and continuous glucose monitoring (CGM)-derived estimates (5, 6). What remains less clearly addressed is how discordance can be linked to the clinical decision being made, the laboratory information needed before a result is trusted, and the metric choice most likely to prevent unsupported diagnosis, intensification, deintensification, or false reassurance.

This review focuses on three anchor settings in which metric choice can plausibly change patient management and in which HbA1c unreliability may arise from both biology and measurement context. In chronic kidney disease (CKD) G4-G5 and dialysis, red-cell turnover, anemia treatment, albumin or protein context, dialysis modality, CGM accuracy, and hypoglycemia risk can coexist. In anemia and altered red-cell turnover, iron deficiency, vitamin B12 or folate deficiency, hemolysis, bleeding, transfusion, erythropoiesis-stimulating agents (ESAs), and active hematologic correction may move HbA1c in different directions. In hemoglobin variants and hemoglobinopathies, biological non-interpretability needs to be distinguished from method-specific analytical interference before a result is accepted, qualified, or rejected.

These settings were selected because they combine clinical frequency or consequence, well-described failure mechanisms, and direct risk of overtreatment, undertreatment, diagnostic delay, or therapeutic inertia (7–9).

This narrative review proposes a clinical-laboratory interpretive framework and is not a systematic review, meta-analysis, guideline, expert consensus statement, or treatment algorithm.

The framework is designed to help readers ask five recurring questions: what decision is being made, why HbA1c may mislead, what needs checking first, which adjunct metric may help, and which clinical action requires confirmation before it is taken. Its intended contribution is not another catalogue of HbA1c limitations, but a decision-linked synthesis for selected high-risk scenarios in which misreading a marker can lead to overtreatment, undertreatment, diagnostic delay, false reassurance, or therapeutic inertia (6, 10).

The review used an iterative, structured narrative approach rather than a systematic database-screening protocol. PubMed/MEDLINE searches were run through NCBI E-utilities on May 28, 2026; related Web of Science-style strings were used for overlap-risk and citation-chaining checks rather than formal systematic screening.

The PubMed strategy combined HbA1c or glycated hemoglobin with terms for discordance, mismatch, reliability, red-cell turnover, assay interference, CKD or dialysis, anemia or transfusion, hemoglobin variants or hemoglobinopathies, CGM or GMI, SMBG or plasma glucose, glycated albumin, fructosamine, 1,5-AG, and glycation-gap or HGI concepts. A recent-review overlap query was limited to 2023 onward; the other targeted searches were not treated as an exhaustive date- or language-restricted corpus.

The search log recorded 1,746 unique PubMed candidates after PMID-level deduplication. Sources were retained when they directly informed HbA1c reliability, adjunct metric interpretation, assay interference, or clinical decision risk in the selected settings. Records were not used for manuscript claims when they addressed only generic biomarker performance, general CGM management without HbA1c-discordance relevance, peripheral populations outside the review scope, or non-glycemic laboratory topics. Case reports and method-limited studies were treated as illustrative or boundary evidence.

Full PubMed search strings, the recent-review overlap-control query, citation-chaining approach, and topic-specific inclusion and exclusion logic are provided in Supplementary Methods.

**Figure 1:**
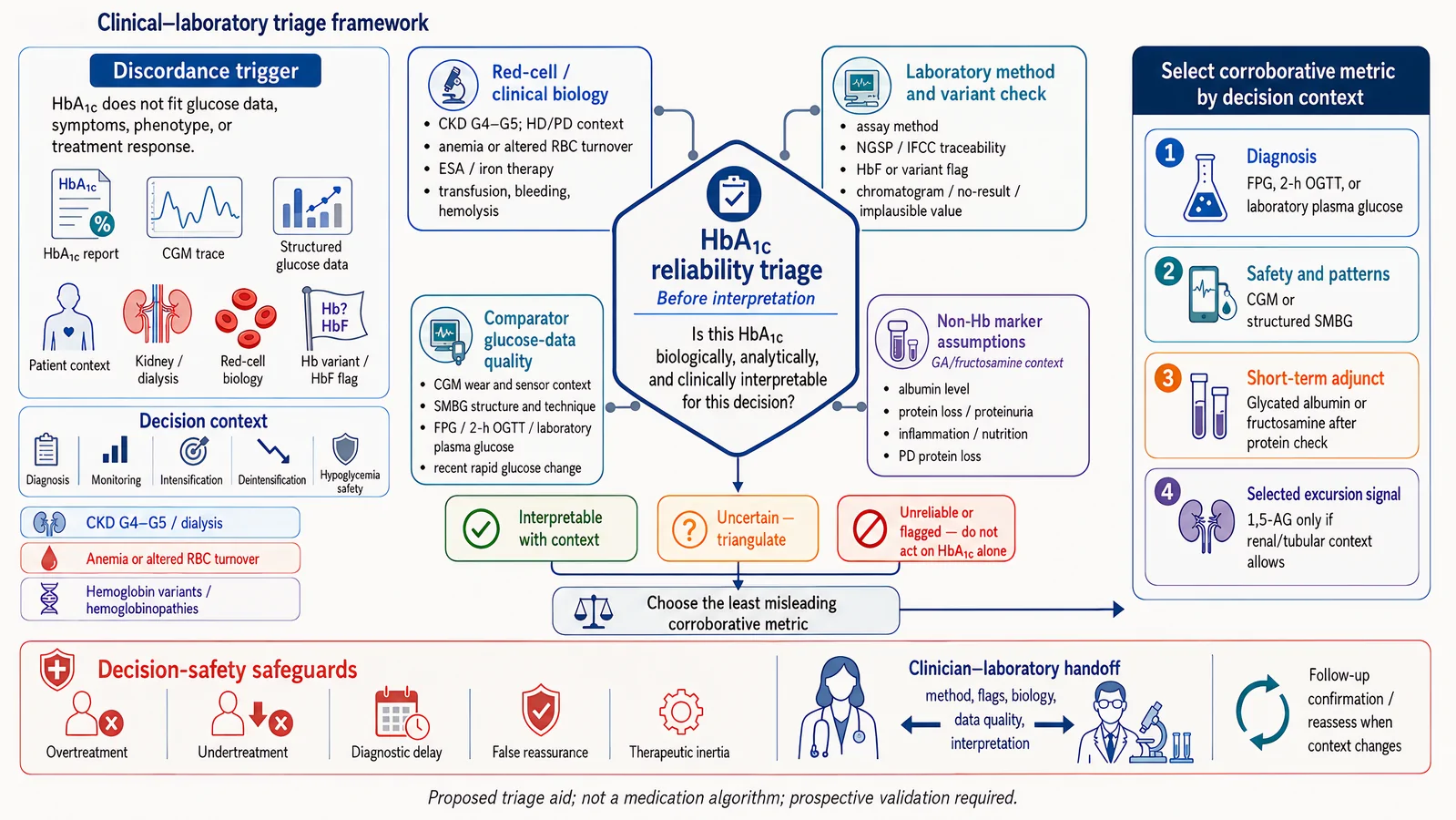
Clinical-laboratory framework for discordant glycemic assessment. Conceptual clinician-laboratory framework for diabetes care when HbA1c may not reliably represent glycemic exposure. The figure begins with a discordance signal and separates the main checks needed before interpretation: clinical decision context, red-cell and kidney biology, assay and hemoglobin-variant context, comparator glucose-data quality, albumin and protein assumptions for non-hemoglobin markers, and safeguards against unsupported diagnosis, intensification, deintensification, false reassurance, or therapeutic inertia. HbA1c, hemoglobin A1c; CKD, chronic kidney disease; HD, hemodialysis; PD, peritoneal dialysis; RBC, red blood cell; ESA, erythropoiesis-stimulating agent; NGSP, National Glycohemoglobin Standardization Program; IFCC, International Federation of Clinical Chemistry and Laboratory Medicine; HbF, fetal hemoglobin; CGM, continuous glucose monitoring; SMBG, self-monitoring of blood glucose; FPG, fasting plasma glucose; OGTT, oral glucose tolerance test; GA, glycated albumin; 1,5-AG, 1,5-anhydroglucitol. The figure reflects synthesis across the four recurring interpretive domains described in the approach section and is intended to support interpretation and communication; it does not specify diagnostic thresholds or medication changes.

The framework was then constructed through an author-level grouping process. Two authors (TW and ZG) grouped retained sources by the clinical question they informed and by four interpretive domains: biological plausibility of HbA1c, method and reporting plausibility, comparator glucose-data quality, and the consequence of acting on the result. Uncertain placements and discrepancies were discussed with WC, whose role included laboratory-medicine interpretation, before the framework was converted into **Figure 1**; **Table 1**.

**Table 1:** Scenario-specific interpretation of HbA1c discordance.

Scenario	Why HbA1c may mislead	Check first	Adjunct information that may help	Action to avoid without confirmation
CKD G4-G5 before dialysis	Anemia, altered red-cell turnover, kidney-disease physiology, and treatment exposures may separate HbA1c from glucose exposure	CKD stage, anemia status, ESA/iron exposure, recent glucose change, albumin/protein context	Laboratory glucose or OGTT for diagnosis; CGM or structured SMBG for monitoring; GA/fructosamine only after protein context check	Applying a generic HbA1c correction or changing therapy from HbA1c alone
Hemodialysis	Shortened red-cell survival, anemia treatment, blood loss, dialysis timing, and hypoglycemia risk can distort longer-window interpretation	Dialysis schedule, ESA/iron timeline, symptoms, insulin use, device context	CGM or structured SMBG for hypoglycemia and variability; selected GA/fructosamine with albumin/protein caveat	Deintensifying therapy or dismissing hyperglycemia because HbA1c is low
Peritoneal dialysis	PD has glucose dialysate exposure and protein/albumin issues that differ from hemodialysis	PD modality, dialysate exposure, residual kidney function, albumin/protein loss	CGM/SMBG for glucose pattern; protein-glycation markers only if protein context allows	Importing hemodialysis assumptions into PD
Iron deficiency or deficiency treatment	HbA1c may be elevated relative to glucose and may fall after correction without true glycemic improvement	CBC, iron indices, treatment timeline, direct glucose data	Laboratory glucose for diagnosis; CGM/SMBG for monitoring if needed	Intensifying therapy automatically from a high HbA1c
B12/folate deficiency or mixed anemia	Evidence supports misleading elevation in selected contexts, but effects are not uniform	Deficiency status, mixed anemia features, treatment timing	Direct glucose corroboration; repeat interpretation after context stabilizes when appropriate	Treating all deficiency states as a single predictable HbA1c bias
Hemolysis, bleeding, transfusion, ESA therapy, or active red-cell recovery	Shortened, replaced, or rapidly changing red-cell exposure can make HbA1c falsely low or unstable	Hemolysis/bleeding/transfusion history, ESA or iron timeline, reticulocyte context where available	Laboratory glucose/OGTT for diagnosis; CGM or SMBG for monitoring	Interpreting a falling HbA1c as true glycemic improvement
Common heterozygous HbS/HbC/HbD/HbE traits	Analytical interference is method-specific; biology and population context may still matter	Variant identity, HbA1c method, report flags, local method performance	Method-qualified HbA1c if supported; glucose data if discordant	Blanket acceptance or rejection of HbA1c
Sickle cell disease, compound hemoglobinopathy, absent/reduced HbA, high HbF, or rare variant	HbA1c may be biologically uninterpretable, analytically interfered with, or both	HbA/HbF context, transfusion or hydroxyurea exposure, abnormal profile/no result	Glucose-based diagnosis; CGM/SMBG for monitoring; selected non-hemoglobin markers if suitable	Diagnosing or excluding diabetes by HbA1c alone
HbA1c-GMI or HbA1c-glucose discordance without an obvious cause	Either the HbA1c side or the comparator glucose side may be flawed	CGM wear/data quality, recent glucose change, method, anemia/variant/CKD/protein context	Recheck direct glucose data and HbA1c plausibility before therapy change	Treating GMI as a direct HbA1c correction

CKD, chronic kidney disease; ESA, erythropoiesis-stimulating agent; OGTT, oral glucose tolerance test; CGM, continuous glucose monitoring; SMBG, self-monitoring of blood glucose; GA, glycated albumin; PD, peritoneal dialysis; HbF, fetal hemoglobin; GMI, glucose management indicator. Evidence basis: **Table 1** summarizes guideline and laboratory-standard safeguards, multi-study scenario evidence, and author interpretive synthesis where direct outcome-validated decision pathways are not available.

Throughout this review, statements grounded in guidelines, laboratory standards, or consistent multi-study evidence are presented as evidence-supported interpretations; where evidence is heterogeneous or indirect, the text reflects author synthesis intended to support corroboration rather than prescribe management. This process generated an interpretive aid for clinician-laboratory communication; it was not an expert consensus process or a prospectively validated algorithm.

## 2 Before interpreting HbA1c: a practical reliability check

Clinically important HbA1c-glucose discordance is best treated first as a reliability question. The issue is not whether to abandon HbA1c, but whether the current value is interpretable for this patient, with this assay, these comparator data, and this clinical decision. Broad mismatch mechanisms, HbA1c-GMI discordance, and CGM-derived metrics each illuminate part of the problem; none provides a universal correction (5, 6).

The first step is to define the decision. Diagnosis, longitudinal monitoring, intensification, deintensification, and safety surveillance tolerate uncertainty differently. If the question is diagnosis and HbA1c is unreliable, diagnostic confirmation is better supported by laboratory plasma glucose criteria. If the question is hypoglycemia or glycemic variability, direct glucose data are more informative than a longer-window glycation marker. If the question is longitudinal exposure in a red-cell-discordant state, a protein-glycation marker may help only after albumin and protein turnover are considered. These distinctions prevent adjunct metrics from being used outside the decision they can answer (1, 2, 7).

The second step is to check the comparator. A gap between HbA1c and GMI may reflect HbA1c-side biology or assay interference, but it may also reflect CGM wear, sensor performance, dialysis-period accuracy, recent rapid glucose change, or the population-derived equation that converts mean sensor glucose into GMI. GMI is therefore a signal that disagreement exists, not a laboratory correction for HbA1c. When the disagreement is large enough to influence care, a lower-risk interpretation examines both sides of the comparison before therapy is changed (6, 11).

The third step is to make the laboratory context explicit. HbA1c standardization and traceability are essential, but they do not remove patient-specific unreliability. In high-risk settings, the laboratory information needed may include the HbA1c method, point-of-care context, National Glycohemoglobin Standardization Program (NGSP) or International Federation of Clinical Chemistry and Laboratory Medicine (IFCC) traceability, hemoglobin variant or fetal hemoglobin (HbF) flags, chromatogram or electropherogram abnormalities where available, and implausible or no-result outputs. Method-specific interpretation matters: the question is not whether an entire assay class is always valid or invalid, but whether the reported number is plausible in the current biological and analytical context (2, 12).

Point-of-care HbA1c literature further supports treating device quality, operator context, traceability, and variant interference as reliability inputs rather than background details (13, 14). CKD method-comparison data reveal that kidney-disease biology and assay method can intersect, so an implausible HbA1c in advanced CKD is a reason for both clinical and laboratory review (15).

The final step is to select a corroborating source of information that matches the decision. Fasting plasma glucose, 2-hour oral glucose tolerance test (OGTT) glucose, or clinically appropriate laboratory plasma glucose supports diagnosis when HbA1c is unreliable. CGM and structured self-monitoring of blood glucose (SMBG) support monitoring, treatment safety, and pattern recognition. Glycated albumin (GA) and fructosamine can support shorter-window monitoring when red-cell markers are unreliable, but only if albumin and protein context do not undermine them. This reliability check is used in the scenario sections that follow (1, 16).

## 3 CKD G4-G5 and dialysis: separate kidney-failure contexts before choosing a metric

Advanced CKD and dialysis are not a single HbA1c-discordance scenario. HbA1c interpretation may be affected by red-cell turnover, anemia, ESA or iron exposure, dialysis modality, uremic milieu, albumin and protein balance, and hypoglycemia risk. The practical question is not whether HbA1c is always wrong in CKD, but which concurrent conditions make the current result unreliable as a stand-alone basis for decisions (7, 17). CKD-focused reviews and biomarker-comparison studies support this plural interpretation: HbA1c, GA, fructosamine, and CGM can each be informative, but their biases and outcome links differ across CKD stage, dialysis exposure, anemia, carbamylation, and albumin context (18–20).

In CKD G4-G5 before dialysis, anemia and kidney-disease physiology can already separate HbA1c from glucose exposure. A non-dialysis CKD patient with anemia and a discordant GMI is poorly served by applying a generic correction factor to HbA1c. A cautious approach is to verify the glucose pattern, red-cell status, recent treatment exposures, and the decision at stake. This caution applies particularly to advanced CKD accompanied by anemia or related disturbances and is not intended to be generalized as a single rule for all earlier CKD stages (7, 21).

Hemodialysis adds further sources of discordance. Shortened red-cell survival, anemia, ESA exposure, iron therapy, blood loss, and dialysis-related physiology can make HbA1c lower than concurrent glucose exposure or GA in many studied cohorts, although the magnitude and direction depend on population, treatment timeline, comparator metric, and dialysis setting. A falling or low HbA1c during active anemia treatment or dialysis is better treated as provisional until it is checked against direct glucose data or a context-appropriate adjunct marker (22–24).

CGM can be especially useful in hemodialysis because it shows interdialytic and dialysis-day patterns, time below range, variability, and symptoms that HbA1c compresses or misses. It is best framed as a monitoring and safety tool whose interpretation depends on device, setting, wear, comparator method, and dialysis timing (25, 26). Recent device- and setting-specific accuracy studies support attention to sensor performance and dialysis setting (27–29), biomarker comparisons support biochemical corroboration (30–32), and CGM-metric studies reinforce the relevance of dialysis-specific patterns and safety metrics (33–35).

Peritoneal dialysis (PD) requires its own interpretive approach. PD may involve glucose-containing dialysate exposure, residual kidney function differences, body-composition and fluid issues, and albumin or protein loss that can alter both glucose patterns and protein-glycation markers. Because direct evidence in PD is more limited than in hemodialysis, interpretation is better kept modality-specific, without extrapolating hemodialysis-based assumptions to all kidney-failure patients (36–38).

Long-term CGM work in PD and end-stage kidney disease, including low-HbA1c or burnt-out diabetes contexts, supports asking whether an acceptable HbA1c is masking dysglycemia or hypoglycemia rather than importing hemodialysis assumptions into all kidney-failure patients (39–41).

GA and fructosamine are attractive in CKD because they do not depend on hemoglobin glycation over the red-cell lifespan. Their independence from red cells is not independence from biology. Albumin concentration, protein turnover, inflammation, nutrition, proteinuria, nephrotic-range losses, and PD-related protein loss can all distort interpretation. A CKD-focused approach is better not to replace one unexamined surrogate with another. The goal is to choose the metric whose assumptions are least affected for the patient and the decision (38, 42, 43).

Prognostic associations involving GA or the GA/HbA1c ratio in CKD and dialysis make these markers clinically relevant, but they do not by themselves show that GA-guided treatment improves outcomes in every CKD subgroup (44).

The clinical reason to separate CKD subgroups is harm prevention. A falsely reassuring HbA1c may promote deintensification, therapeutic inertia, or missed hyperglycemia, whereas a falsely high adjunct marker may encourage unnecessary intensification. A seemingly acceptable HbA1c can also obscure hypoglycemia and variability in insulin-treated dialysis patients. From an interpretive standpoint, safer decision-making usually requires direct glucose data, explicit dialysis modality, red-cell and albumin context, and a qualified interpretation of any adjunct marker. This remains an interpretive approach for risk recognition and corroboration rather than a therapeutic pathway (7, 10).

## 4 Anemia and altered red-cell turnover: direction, timeline, and decision matter

Anemia is not a single HbA1c confounder. Its effect depends on red-cell survival, erythropoiesis, the dominant deficiency or destruction process, recent treatment, transfusion, and whether the circulating red-cell population is stable or changing. A low hemoglobin concentration alone is an incomplete signal. The more relevant question is whether red cells have been exposed to glucose in a way that makes the current HbA1c interpretable for diagnosis, monitoring, intensification, or deintensification (8, 45).

Iron deficiency illustrates why subtype and timeline matter. Several studies and syntheses support that iron deficiency anemia can raise HbA1c relative to glucose and that iron replacement can lower HbA1c without an equivalent glycemic change. However, the evidence is not perfectly consistent across populations, anemia definitions, baseline glycemia, and treatment conditions. The appropriate conclusion is therefore practical rather than formulaic: suspected or treated iron deficiency is a reason for glucose corroboration before HbA1c is used to escalate therapy or reassure the patient (8, 46, 47). Evidence across iron deficiency, GA, and mixed anemia phenotypes supports the same subtype-specific framing; anemia can change HbA1c interpretation, but the direction and the behavior of adjunct markers depend on the hematologic state (48–50).

Vitamin B12 and folate deficiency states create a related but not identical problem. Available evidence supports misleading HbA1c elevation in selected deficiency states and reduction after correction without a matching fasting-glucose change. That is enough to justify checking deficiency status when HbA1c and glucose data disagree, but not enough to create one rule for all megaloblastic, nutritional, or mixed anemia states. If HbA1c is high during active deficiency or early correction, treatment intensification is better based on direct glucose evidence whenever clinical urgency allows (51, 52).

The opposite pattern can occur when red-cell exposure is shortened, replaced, or rapidly changing. Hemolysis, bleeding, transfusion, ESA therapy, iron therapy, and recovery from anemia can make HbA1c falsely low, unstable, or difficult to trend because the measured hemoglobin pool no longer represents the usual glycation window. In these settings, HbA1c is most dangerous when interpreted as improvement or reassurance (8, 53). Transfusion-dependent and hemoglobinopathy-adjacent evidence supports using glucose-based diagnosis and direct monitoring rather than HbA1c alone (54, 55), whereas selected case reports and non-diabetic or highly selected studies remain illustrative rather than definitive (56, 57). Evidence from aplastic anemia and related settings also supports using non-hemoglobin markers only as context-aware adjuncts, not universal substitutes (58).

The practical consequence is a need for decision-specific corroboration. A falsely elevated HbA1c in a deficiency state can lead to overtreatment, while a falsely low or rapidly falling HbA1c after transfusion, hemolysis, ESA therapy, or iron correction can cause false reassurance, diagnostic delay, undertreatment, or inappropriate deintensification. A lower-risk clinical choice is to identify the anemia subtype and treatment timeline, ask whether red-cell exposure is stable, use laboratory glucose or OGTT for diagnostic questions, and reserve CGM, SMBG, GA, or fructosamine for monitoring when their own assumptions are not affected. Therefore, HbA1c interpretation after anemia treatment, transfusion, or erythropoiesis-stimulating therapy is best individualized rather than based on a universal waiting interval or fixed correction factor (8, 53).

## 5 Hemoglobin variants and hemoglobinopathies: distinguish biology from method interference

Hemoglobin variants and hemoglobinopathies can be approached through two questions that are often conflated. The first is biological: does the patient have altered red-cell survival, absent or reduced HbA, transfusion exposure, hydroxyurea-modified erythropoiesis, or compound hemoglobinopathy that makes HbA1c a poor marker of glycemic exposure even if a number is reported? The second is analytical: does a variant or HbF interfere with the specific HbA1c method so that the result is biased, flagged, or not reportable? (2, 59).

Because analytical interference is method-specific, the useful laboratory question is not simply whether a variant exists. It is which variant is present or suspected, which method was used, whether the result is reportable, and whether the chromatogram, electropherogram, abnormal fraction, no-result output, or implausible value suggests interference. High-performance liquid chromatography, capillary electrophoresis, immunoassay, enzymatic, boronate affinity, and mass-spectrometry-based approaches can behave differently across variants and study settings. This supports method identification and current method-specific checking, not universal assay-class ranking (9, 60, 61).

Method-comparison studies across multiple variants and systems reinforce that a finding from one platform, variant set, or population is not generalizable to all analyzers (62–64). In practice, variant-associated HbA1c interpretation is strengthened by consulting current method-specific interference resources, including the NGSP HbA1c Assay Interferences table, manufacturer product inserts, and local validation data before the result is used for clinical decisions (9, 60–64).

Common heterozygous traits require method-aware interpretation rather than automatic rejection. For HbS, HbC, HbD, or HbE trait, selected modern methods may show limited clinically important interference in some studies, but trait status, population background, local method performance, and the decision context still matter. A method-verified HbA1c may be acceptable when red-cell biology is stable and the assay is suitable; a discordant or flagged result warrants corroboration with glucose data before diagnosis or treatment changes (59, 60, 65).

Sickle cell disease, compound hemoglobinopathy, absent or markedly reduced HbA, high HbF, heavy transfusion exposure, and hydroxyurea-treated erythropoiesis require a different interpretive approach. In these settings, the problem may be biological, analytical, or both. HbA1c can be misleading even when an analyzer technically produces a result. Diagnostic confirmation is better supported by glucose-based testing when HbA1c is biologically uninterpretable, and monitoring is better anchored in CGM, SMBG, or selected non-hemoglobin markers only after their limitations are considered (66–68).

Laboratory flags are clinically useful because they can prevent forced interpretation of a potentially misleading number. A no-result HbA1c, abnormal chromatogram, unexpected hemoglobin fraction, double peak, variant tail, or implausible value is a reason for clinician-laboratory communication rather than routine treatment action. Population studies support the clinical relevance of variant-associated flags and discordance (69–71), whereas rare-variant reports are valuable warning examples but are best framed as method- and setting-specific illustrations rather than prevalence estimates or analyzer rankings (72–74).

The practical handoff is: which variant or flag, which method, what red-cell context, and what decision? If the main issue is method interference in a biologically stable trait, a method-qualified HbA1c or alternate validated method may still be useful. If the issue is absent HbA, unstable erythropoiesis, transfusion, sickle cell disease, or compound hemoglobinopathy, direct glucose assessment is usually the stronger anchor for diagnosis and safety monitoring. **Table 2** summarizes these distinctions for practical interpretation (2, 9, 75).

**Table 2:** Hemoglobin variant and assay-method interpretation.

Context	Main reliability issue	Laboratory question	Clinical interpretation	Boundary
Common heterozygous HbS, HbC, HbD, or HbE trait	Usually not absent HbA, but assay interference and population context may matter	Which method was used, and is this variant supported by current method-specific information?	HbA1c may be usable if method and clinical context support it; corroborate if discordant	No blanket acceptance or rejection
Sickle cell trait	Trait needs to be distinguished from sickle cell disease	Does local method performance support reporting in this population?	Use HbA1c only with method awareness; verify discordant results with glucose data	Platform- and population-dependent
Sickle cell disease or compound hemoglobinopathy	Red-cell survival, HbA absence/reduction, transfusion, and hydroxyurea may make HbA1c biologically uninterpretable	Is there enough HbA and stable red-cell biology for HbA1c to mean glycemic exposure?	Prefer glucose-based diagnosis and direct monitoring	Do not merge trait with disease
High HbF or abnormal hemoglobin profile	Biological and analytical effects may coexist	Is there a flag, abnormal peak, no result, or method-specific HbF limitation?	Do not use a universal HbF threshold; use direct glucose or qualified alternatives	Requires method-specific support
Rare variant, double peak, variant tail, or implausible value	Variant-specific behavior may be unknown	Can the lab explain the result or recommend an alternate validated method?	Treat as a handoff trigger; avoid forced numeric interpretation	Case reports are illustrative, not prevalence estimates
G6PD deficiency or other red-cell survival disorder	Red-cell biology rather than structural hemoglobin interference may dominate	Is shortened red-cell survival plausible?	Include in discordance workup and use glucose corroboration	Adjacent red-cell disorder, not an assay-class claim

HbA, adult hemoglobin A; HbF, fetal hemoglobin; G6PD, glucose-6-phosphate dehydrogenase. Method-specific interpretation is best checked against current NGSP HbA1c assay-interference information, manufacturer instructions, and local laboratory validation before clinical use. Evidence basis: **Table 2** is based on method-specific hemoglobin-variant literature, laboratory-standard principles, the NGSP assay-interference resource, and author synthesis for the clinician-laboratory handoff.

## 6 Adjunct glycemic metrics as cross-cutting tools: use hierarchy, not interchangeability

**Figure 2:**
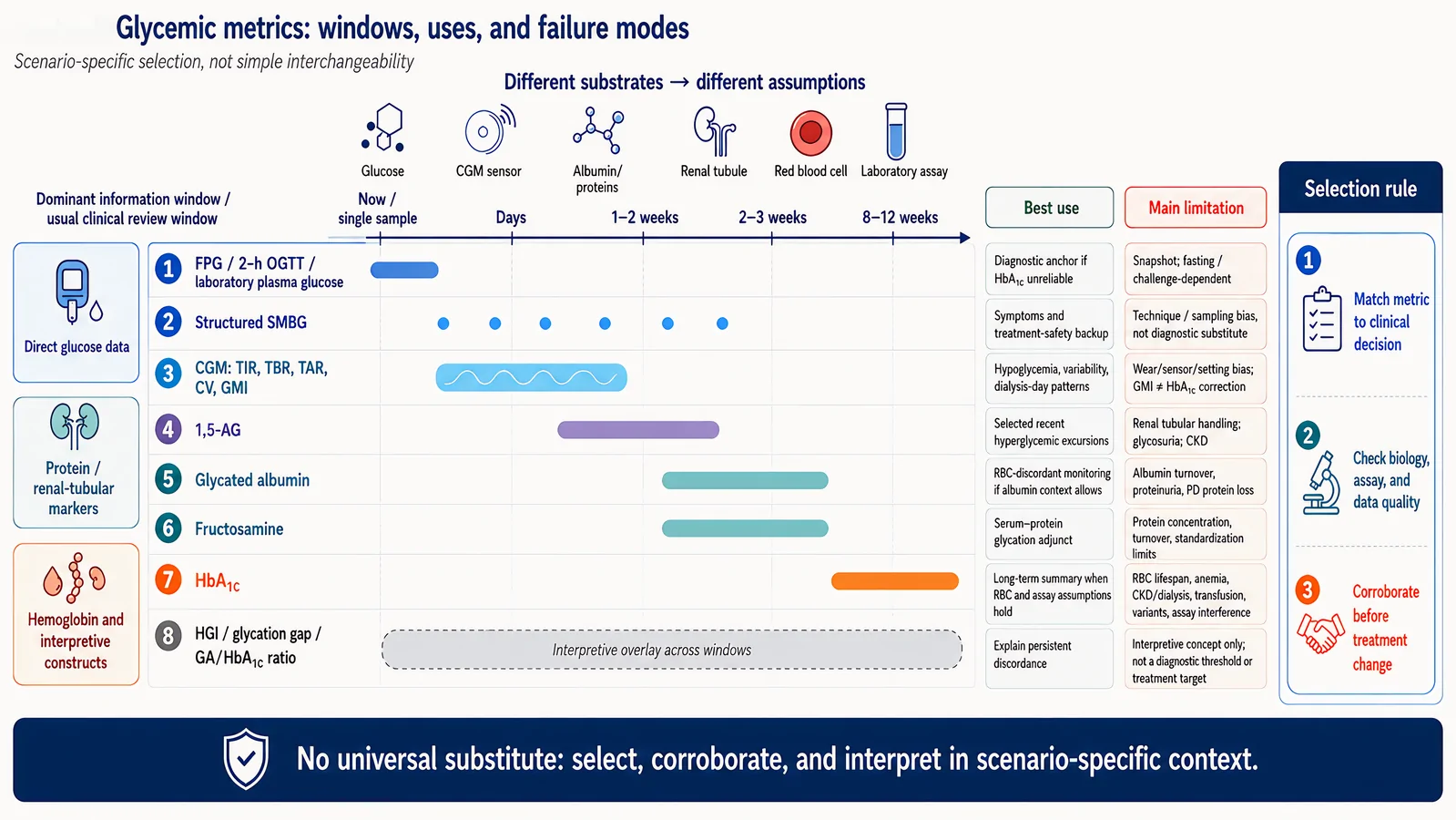
Glycemic metrics: windows, uses, and failure modes. Summary of how common glycemic metrics differ by exposure window, substrate, data dependency, and major failure mode. Direct glucose measurements and OGTT provide diagnostic anchors when HbA1c is unreliable; CGM and structured SMBG support monitoring, hypoglycemia detection, and pattern recognition; GA and fructosamine provide shorter-window protein-glycation information when albumin and protein turnover are interpretable; 1,5-AG is limited by renal tubular and glycosuria physiology; and hemoglobin glycation index, glycation gap, or GA/HbA1c ratio concepts are interpretive constructs rather than treatment targets. Abbreviations are as defined for **Figure 1**. The figure emphasizes scenario-specific selection rather than interchangeability among markers.

Adjunct glycemic metrics are discussed here as cross-cutting tools for the anchor scenarios rather than as a stand-alone biomarker catalogue. This section does not expand the disease scope beyond the three anchor settings; it summarizes adjunct tools used across them. The exposure windows, decision roles, and limitations of these metrics are summarized in **Figure 2** and **Table 3**. They are best treated as conditional tools rather than automatically valid substitutes when HbA1c becomes unreliable. Each answers a different clinical question, covers a different time window, and depends on different biological or technical assumptions. The core issue is choosing the metric that best fits the decision while making its failure modes visible (42, 76).

**Table 3:** Adjunct glycemic metrics, uses, and limitations.

Metric	Primary role	Best use when HbA1c is unreliable	Main limitation	Not appropriate as
Laboratory plasma glucose or OGTT	Diagnostic confirmation	Diagnosis when HbA1c is biologically or analytically unreliable	Snapshot or challenge-dependent result	Longitudinal exposure summary by itself
CGM metrics including TIR, TBR, TAR, CV, mean glucose, and GMI	Direct glucose-pattern monitoring	Hypoglycemia, variability, dialysis-day patterns, symptoms, treatment safety	Wear, sensor accuracy, setting, dialysis timing, and GMI translation	Laboratory diagnostic substitute
Structured SMBG	Pragmatic direct glucose backup	Symptom checks, treatment safety, CGM backup, resource-limited monitoring	Sparse sampling, timing, technique, nocturnal gaps	Diagnostic replacement for laboratory criteria
Glycated albumin	Shorter-window protein glycation	Red-cell-discordant monitoring when albumin/protein context is acceptable	Albumin concentration, turnover, protein loss, inflammation, nutrition, PD	Universal HbA1c replacement threshold
Fructosamine	Shorter-window serum-protein glycation	Adjunct monitoring when HbA1c is unreliable and protein context is acceptable	Protein concentration, turnover, standardization limitations	Stand-alone diagnostic anchor
1,5-AG	Selected recent excursion signal	Narrow adjunct outside renal/tubular-confounded contexts	Renal tubular handling and glycosuria physiology	Primary CKD metric or treatment determinant
HGI, glycation gap, GA/HbA1c ratio	Interpretive explanation of persistent mismatch	Research or explanatory context for discordant markers	Model and comparator dependence; risk of overinterpretation	Routine treatment target

OGTT, oral glucose tolerance test; CGM, continuous glucose monitoring; TIR, time in range; TBR, time below range; TAR, time above range; CV, coefficient of variation; GMI, glucose management indicator; SMBG, self-monitoring of blood glucose; PD, peritoneal dialysis; 1,5-AG, 1,5-anhydroglucitol; HGI, hemoglobin glycation index; GA, glycated albumin. Evidence basis: **Table 3** summarizes guideline/laboratory diagnostic principles, direct glucose-monitoring evidence, multi-study biomarker evidence, and author synthesis where adjunct metrics remain interpretive rather than validated treatment targets.

Direct glucose data sit at the center of most high-discordance decisions. CGM is especially valuable for recent exposure, hypoglycemia, glycemic variability, dialysis-day patterns, symptoms, and treatment safety. Time in range, time below range, time above range, mean sensor glucose, coefficient of variation, and GMI reveal dimensions that HbA1c compresses or misses. They still require attention to wear time, data completeness, sensor performance, setting, dialysis timing, and rapid recent change (1, 77). Structured SMBG remains useful when CGM is unavailable or questionable, but it is not a substitute for laboratory diagnostic testing. When the question is diagnosis and HbA1c is unreliable, fasting plasma glucose, 2-hour OGTT glucose, or clinically appropriate laboratory plasma glucose remains the diagnostic anchor (1, 16).

GA and fructosamine are the main non-hemoglobin glycation markers discussed in this review. Their shorter window and independence from red-cell lifespan make them attractive in red-cell-discordant states, including some CKD, anemia, transfusion, or hemoglobinopathy contexts.

Their limitations are equally central: albumin concentration, protein turnover, protein loss, nephrotic-range proteinuria, PD, inflammation, liver or thyroid context, nutrition, and standardization can all affect interpretation (42, 43).

Studies linking GA or fructosamine to CGM patterns or cardiovascular outcomes support clinical relevance, but they support adjunctive interpretation rather than universal replacement thresholds (78–80). Population screening work combining HbA1c with GA or 1,5-anhydroglucitol (1,5-AG) does not remove the need for glucose-based diagnostic confirmation when HbA1c reliability is compromised (81).

1,5-AG is best kept in a narrower role. It can provide information about recent hyperglycemic excursions in selected settings, but it depends on renal tubular handling and glycosuria physiology. When CKD or renal-threshold physiology is central to the discordance problem, 1,5-AG is difficult to interpret and is a weak basis for diagnosis or treatment decisions (76, 82).

Hemoglobin glycation index, glycation gap, and GA/HbA1c ratio concepts can help explain persistent marker disagreement or inter-individual glycation differences, but they are interpretive constructs rather than routine clinical targets. Evidence involving alcohol exposure and GA/HbA1c ratio behavior illustrates marker-specific discordance mechanisms (83–85), while CGM-derived range metrics strengthen the need for an interpretive layer without turning these constructs into medication thresholds (86–88).

## 7 Synthesis: a clinician-laboratory approach to discordant glycemic assessment

The preceding sections converge on a simple principle: discordance is a reason to slow interpretation before making any changes to treatment. The proposed framework begins with a discordance signal, defines the clinical decision, checks HbA1c biology and method plausibility, examines the quality of comparator glucose data, and then selects an adjunct metric that fits the decision. As a proposed interpretive structure, it is intended to make assumptions explicit in the high-risk scenarios reviewed above (6, 7).

The clinician’s contribution is to define the decision and the harm to avoid. Diagnosis, monitoring, intensification, deintensification, and hypoglycemia surveillance are not interchangeable. In CKD and dialysis, the relevant clinical information includes dialysis modality, insulin or hypoglycemia risk, recent ESA or iron therapy, and albumin or protein context. In anemia, it includes subtype, treatment timeline, transfusion, hemolysis, and red-cell stability. In hemoglobin variants, it includes whether the patient has trait, disease, transfusion exposure, or another state that changes red-cell biology (6, 7).

The laboratory contribution is to explain whether the reported HbA1c can be interpreted in the first place. Assay method, traceability, point-of-care status, HbF or variant flags, abnormal chromatogram or electropherogram patterns, and interpretive comments can all affect whether a number is routine, qualified, or uninterpretable. This does not require every laboratory to create a new consultation service; it requires assay and specimen context to be communicated when it materially affects metric choice (2, 9, 75).

The glucose-data contribution is to show exposure and safety directly. CGM interpretation depends on wear, data sufficiency, sensor setting, dialysis timing where relevant, mean glucose, GMI, time in range, time below range, variability, and symptom concordance. SMBG is best judged by structure, timing, technique, and whether it answers the clinical question. Diagnostic uncertainty is better resolved with laboratory glucose testing when HbA1c is unreliable. In high-risk discordance, a treatment change based on one unexamined marker is usually less defensible than triangulation with at least one direct glucose source and an explicitly qualified biomarker (1, 7, 42).

The clinical value of the framework is its harm orientation. A spuriously low HbA1c may produce false reassurance, undertreatment, deintensification, or delayed diagnosis. A spuriously high HbA1c may produce unnecessary intensification. An apparently acceptable HbA1c may hide hypoglycemia or variability in insulin-treated dialysis patients. The framework therefore asks which action requires confirmation and what minimum evidence is needed before that action proceeds (10, 17, 89).

The framework is best interpreted within the limits of guideline principles, laboratory-method evidence, scenario-specific studies, and treatment-risk logic. It does not specify medication changes, universal follow-up intervals, or outcome-proven superiority of one metric-selection pathway. Its current role is to support clinician-laboratory communication and to define testable research questions (10, 76, 90).

## 8 Discussion: limitations, implementation, and validation needs

This review reframes HbA1c discordance as a decision problem. Measurement error alone is not the main clinical danger; the danger is acting as if a biologically or analytically fragile result were unquestionably interpretable. A practical approach therefore makes five assumptions visible: the patient context, the assay context, the quality of direct glucose data, the assumptions behind adjunct markers, and the immediate diagnostic or treatment decision (6, 10, 42).

Several limitations remain important. This is a narrative review with a proposed interpretive framework, not a systematic review, meta-analysis, guideline, or prospectively validated algorithm. The evidence reviewed here does not support universal GA, fructosamine, 1,5-AG, GMI, hemoglobin glycation index, or glycation-gap cutoffs for all high-discordance populations. It also does not support a single medication algorithm, universal waiting period after anemia treatment or transfusion, or outcome-proven metric hierarchy across CKD, anemia, and hemoglobin-variant settings.

Evidence from modality-specific, method-specific, abstract-level, pediatric, non-diabetic, case-report, or illustrative sources requires cautious interpretation and is not readily generalizable beyond its original context (10, 76, 90).

Future work can test the framework rather than simply expand the list of markers. Prospective studies could evaluate scenario-specific reliability checks in CKD G4-G5, hemodialysis, PD, anemia-treatment states, transfusion-dependent populations, and hemoglobinopathy settings. Adjunct markers need comparison against clinically meaningful outcomes and decisions, not only against HbA1c or each other. Laboratory studies could develop practical reporting standards for method, variant flags, HbF, point-of-care context, implausible values, and interpretive comments. Treatment-safety studies can ask whether triangulated interpretation reduces overtreatment, undertreatment, false reassurance, diagnostic delay, or therapeutic inertia (10, 76, 90).

Implementation will require coordinated but realistic handoffs. Clinicians can identify the decision at risk, symptoms, treatment timeline, dialysis modality, anemia subtype, and transfusion or hemoglobinopathy context. Laboratories can provide method, traceability, variant, HbF, and plausibility information. Glucose data can supply direct exposure and safety patterns. Adjunct markers can then be selected only after their own assumptions are checked. This division of labor is modest enough for practice and specific enough for future validation (1, 5, 42).

In conclusion, HbA1c need not be abandoned, but it is not safe to trust it uncritically. In kidney failure, anemia or altered red-cell turnover, and hemoglobin variants or hemoglobinopathies, the more appropriate question is whether HbA1c is interpretable for the decision being made and which additional metric can corroborate the answer without introducing a different source of error. A decision-oriented clinician-laboratory approach can make discordance visible, reduce unsupported clinical action, and guide the studies needed to validate metric selection in high-risk diabetes care.
