# Supplementary material for: Glycemic assessment when hemoglobin A1c is unreliable: a clinical-laboratory framework for kidney disease, anemia, and hemoglobinopathies
Source: Front Endocrinol (Lausanne). 2026 Sep 1;17:1898713. doi: 10.3389/fendo.2026.1898713 (PMC13574703; doi:10.3389/fendo.2026.1898713)
Supplement: Supplementary file 1 [file DataSheet1.pdf]

Supplementary Methods: Literature Identification, Source Selection, and Framework Construction

Scope

This supplementary methods file supports the narrative review "Glycemic Assessment When Hemoglobin A1c Is Unreliable: A Clinical-Laboratory Framework for Kidney Disease, Anemia, and Hemoglobinopathies." The review was not designed as a systematic review, meta-analysis, scoping review, or guideline. The procedures below describe how candidate literature was identified, narrowed, and synthesized for a structured narrative review.

Search Sources and Date

PubMed/MEDLINE searches were run through NCBI E-utilities on May 28, 2026. Web of Science-style strings were also recorded and used for overlap-risk and citation-chaining checks, but they were not treated as a separate formal systematic screening corpus. Returned records were used to define the literature pool and reviewer-risk controls; manuscript claims were based only on sources that were subsequently retained for direct relevance to HbA1c reliability, adjunct metric interpretation, assay interference, or clinical decision risk.

PubMed Search Strings

Broad Search

```
("Hemoglobin A, Glycosylated"[Mesh] OR HbA1c[tiab] OR "glycated
hemoglobin"[tiab])
AND (discordance[tiab] OR discrepanc*[tiab] OR mismatch[tiab] OR
unreliable[tiab] OR "red blood cell lifespan"[tiab] OR "erythrocyte
turnover"[tiab] OR "assay interference"[tiab])
AND (diabetes[tiab] OR "Diabetes Mellitus"[Mesh])
```

Medium Search

```
(HbA1c[tiab] OR "glycated hemoglobin"[tiab])
AND (discordance[tiab] OR discrepanc*[tiab] OR mismatch[tiab] OR
unreliable[tiab])
AND ("chronic kidney disease"[tiab] OR CKD[tiab] OR dialysis[tiab] OR
anemia[tiab] OR "iron deficiency"[tiab] OR hemolysis[tiab] OR transfusion[tiab]
OR "hemoglobin variant*"[tiab] OR hemoglobinopath*[tiab] OR thalassemia[tiab]
OR "sickle cell"[tiab])
```

Precise Search

```
((HbA1c[tiab] OR "glycated hemoglobin"[tiab])
AND (CGM[tiab] OR "continuous glucose monitoring"[tiab] OR "glucose management
indicator"[tiab] OR GMI[tiab] OR "time in range"[tiab] OR "glycated
albumin"[tiab] OR fructosamine[tiab] OR "self-monitoring blood glucose"[tiab]
OR SMBG[tiab])
AND ("chronic kidney disease"[tiab] OR CKD[tiab] OR dialysis[tiab] OR
anemia[tiab] OR "hemoglobin variant*"[tiab] OR hemoglobinopath*[tiab]))
```

Similar-Review Overlap Search, 2023-Present

```
((HbA1c[tiab] OR "glycated hemoglobin"[tiab])
AND (discordance[tiab] OR discrepanc*[tiab] OR mismatch[tiab] OR "glucose
management indicator"[tiab] OR GMI[tiab])
AND (review[pt] OR review[tiab] OR guideline[tiab] OR consensus[tiab] OR
checklist[tiab] OR framework[tiab]))
AND ("2023/01/01"[dp] : "3000"[dp])
```

Query Results and Candidate Pool

Query ID	Scope	Total records	Retrieved into candidate table	Role
PM-BROAD	HbA1c/glycated hemoglobin plus discordance, mismatch, reliability, or interference in diabetes	470	0	Capacity check only
PM-MEDIUM	HbA1c discordance/mismatch plus CKD, dialysis, anemia, transfusion, hemoglobin variants, thalassemia, or sickle cell terms	73	73	High-specificity seed query
PM-PRECISE	HbA1c plus CGM/GMI/TIR/GA/fructosamine/SMBG plus CKD/anemia/Hb variants	282	282	Metric-selection query
PM-REVIEW-2023	HbA1c discordance/GMI review, guideline, checklist, consensus, or framework since 2023	39	39	Recent overlap-control query
AXIS-CKD-TARGET	CKD/dialysis plus HbA1c, GA, fructosamine, CGM, GMI, or TIR	2058	First 500 by relevance	CKD and dialysis pool
AXIS-ANEMIA-TARGET	HbA1c plus anemia, iron, hemolysis, transfusion, ESA, or red-cell turnover	630	First 500 by relevance	Anemia/red-cell turnover pool

AXIS-HBVAR-TARGET	HbA1c plus hemoglobin variants, hemoglobinopathies, thalassemia, sickle cell, or G6PD	406	406	Hemoglobin-variant pool
ALT-METRICS-TARGET	GA, fructosamine, 1,5-AG, GMI, TIR, glycation gap, or HGI plus diabetes and HbA1c	2536	First 500 by relevance	Adjunct-metric pool

After PMID-level deduplication across focused, review-overlap, axis-specific, and alternative-metric queries, the candidate table contained 1,746 unique PubMed records.

Inclusion Logic

Records were retained when they addressed at least one of the following:

- diabetes or glycemic assessment in a clinically relevant setting;
- HbA1c reliability, discordance, mismatch, method limitation, or interpretive failure;
- high-discordance populations including CKD/dialysis, anemia or altered red-cell turnover, and hemoglobin variants or hemoglobinopathies;
- adjunct or triangulated glycemic metrics including CGM/GMI/TIR, SMBG/plasma glucose, glycated albumin, fructosamine, selected 1,5-AG, glycation gap, or HGI;
- guideline, consensus, laboratory-standard, review, checklist, or overlap-control content that helped define the clinical-laboratory framework.

Exclusion Logic

Records were not used for manuscript claims when they were:

- general diabetes biomarker papers without direct HbA1c reliability, high-discordance, or metric-selection relevance;
- general CGM management papers without HbA1c comparison or discordance-decision relevance;
- pregnancy, pediatric-only, critical illness, liver disease, or race/ethnicity papers unless they clarified a boundary relevant to the review;
- non-diabetes or non-glycemic hemoglobinopathy/laboratory papers without HbA1c interpretation relevance;
- D-level records after title/metadata screening.

Screening Tiers

The initial title/metadata screen used A/B/C/D tiers. A-level records were treated as core candidates requiring full or focused reading. B-level records were retained as support candidates for metric selection, guideline, laboratory-method, or scenario-specific claims. C-level records

were reserve/background sources that could be promoted if a section gap appeared. D-level records were excluded or deferred and were not used as manuscript evidence.

After the first screening pass, 120 records were classified as A-level, 461 as B-level, 255 as C-level, and 910 as D-level. The evidence pool was then narrowed by scenario relevance, source quality, and exact claim support before manuscript use.

Evidence Synthesis and Framework Construction

Retained sources were grouped by the clinical question they informed and by four recurring interpretive domains: biological plausibility of HbA1c, method and reporting plausibility, comparator glucose-data quality, and the consequence of acting on the result. Statements grounded in guidelines, laboratory standards, or consistent multi-study evidence were treated as evidence-supported interpretations. Where evidence was heterogeneous, indirect, method-limited, or based on case reports, the manuscript uses author synthesis and frames those sources as illustrative or boundary evidence rather than as validated clinical recommendations.

The resulting framework is an interpretive aid for clinician-laboratory communication. It is not an expert consensus statement, guideline, prospectively validated diagnostic algorithm, or medication pathway.
